# Anticipatory gene regulation driven by maternal effects in an insect–host system

**DOI:** 10.1002/ece3.1763

**Published:** 2015-11-15

**Authors:** Roberto F. Nespolo, Andrea X. Silva, Christian C. Figueroa, Leonardo D. Bacigalupe

**Affiliations:** ^1^ Instituto de Ciencias Ambientales y EvolutivasFacultad de CienciasUniversidad Austral de ChileValdiviaChile; ^2^ Center of Applied Ecology and Sustainability (CAPES)Facultad de Ciencias BiológicasUniversidad Católica de ChileSantiago6513677Chile; ^3^ AUSTRAL‐omicsFacultad de CienciasUniversidad Austral de ChileValdiviaChile; ^4^ Laboratorio de Interacciones Insecto‐PlantaInstituto de Ciencias BiológicasUniversidad de Talca2 Norte 685TalcaChile; ^5^ Millennium Nucleus Centre in Molecular Ecology and Evolutionary Applications in the AgroecosystemsUniversidad de Talca2 Norte 685TalcaChile

**Keywords:** Adaptive evolution, anticipatory gene regulation, aphids, chemical defenses, maternal effects

## Abstract

Adaptive mechanisms involved in the prediction of future environments are common in organisms experiencing temporally variable environments. One of these is AGR (anticipatory gene regulation); in which differential gene expression occur in an individual, triggered by the experience of an ancestor. In this study, we explored the existence of AGR driven by a maternal effect, in an insect–host system. We analyzed gene expression of detoxifying systems in aphids across two generations, by shifting mothers and offspring from chemically defended to nondefended hosts, and vice versa. Then, we measured fitness (intrinsic rate of increase) and the relative abundance of transcripts from certain candidate genes in daughters, using RT‐qPCR (quantitative reverse‐transcription PCR). We found AGR in most cases, but responses varied according to the system being analyzed. For some pathways (e.g., cathepsins), the experience of both mothers and offsprings affected the response (i.e., when both, mother and daughter grew in the defended host, the maximum response was elicited; when only the mother grew in the defended host, an intermediate response was elicited; and when both, mother and daughter grew in a nondefended host, the response was undetectable). In other cases (esterases and GSTs), gene over‐expression was maintained even if the daughter was transferred to the nondefended host. In spite of these changes at the gene‐regulatory level, fitness was constant across hosts, suggesting that insects keep adapted thanks to this fluctuating gene expression. Also, it seems that that telescopic reproduction permits aphids to anticipate stressful environments, by minute changes in the timing of differential gene expression.

## Introduction

Perhaps the most important paradigm‐shift in evolutionary biology after the genomics revolution is the understanding of how phenotypes read genotypes in populations (Rose and Oakley 2007; Whitfield 2008; Travisano and Shaw 2013). Classic population genetic theory defined populations as relatively fixed admixtures of genes packed in organisms, where genes are read to generate phenotypes (Whitfield 2008). This permitted to develop tractable models of adaptive evolution of genes and phenotypes in populations (Hartl and Clark 1997; Roff 1997). However, during the last two decades, researchers have shown an enormous number of possible combinations of traits produced by a given genotype, due to modulations of gene‐expression patterns (Oleksiak et al. 2002; Wang et al. 2009). In fact, today it is well accepted that the subtlest environmental stimulus contribute to phenotypic polymorphism, because of cis‐ and trans‐regulatory changes contribute to divergent gene expression and thus to divergent phenotypes (Hoekstra and Coyne 2007; Wittkopp and Kalay 2012; Harpur et al. 2014).

Humans have tried to eradicate pest insects since the origin of domestication of plants and animals. Documented use of synthetic insecticides dates back to 1939, with dichlorodiphenyltrichloroethane (DDT) and progressed exponentially until today (Denholm et al. 2002). The progressive and rapid appearing of insecticide resistance in pest populations is one of the many examples of fast adaptive change, which is explained in part by preexistent mutations conferring resistance to naturally occurring xenobiotics (Hartley et al. 2006). In fact, most eukaryotic cells respond to chemical stressors by seemingly similar detoxifying mechanisms (Feyereisen 1999; Ffrench‐Constant et al. 2004; Raza 2011).

Classically, invertebrates have been shown to respond to stressful environments (i.e., insecticides, plant allelochemicals) by a rapid adaptation through directional selection of individuals carrying insensibility point mutations (e.g., *kdr*,* super‐kdr*,* rdl*) and/or individuals carrying duplications of certain genes (e.g., P‐450 enzymes, esterases) (Silva et al. 2012a,b) that are spread in populations. This has generated complex coevolutionary trends between pest insects and their host plants particularly in agroecosystems, as insect herbivores have to deal with the allelochemicals produced by their hosts (Castaneda et al. 2010) and the insecticides sprayed for pest control (Denholm et al. 2002).

As bet‐hedging strategy, insect herbivores can quickly change the expression of detoxification genes (i.e., stochastic switching of gene expression; leading to phenotypically plastic responses), whose coded proteins can facilitate excretion, degradation, or sequestration of toxic compounds (Ffrench‐Constant et al. 2004; Silva et al. 2012b). Four of the most important detoxifying enzymatic mechanisms include Cath‐b (cathepsin B), carboxylesterases (EST), GSTs (glutathione S‐transferases) and cytochromes P‐450 (CYPs) (Silva et al. 2012a,b). Cath‐b (EC 3.4.22.0) is a cysteine endopeptidase with broad specificity for peptide bonds, which is directly involved in the hydrolysis of toxic proteins of dietary items (Koo et al. 2008). Esterases (EC 3.1.1.‐) are a general and diversified group of wide specificity hydrolases involved in the degradation of xenobiotics containing carboxylic esters (Dmitryjuk et al. 2014). GSTs (EC 2.5.1.18) are a highly diversified family of enzymes of broad specificity involved in phase II metabolic detoxification that catalyzes the conjugation of reduced glutathione (GSH) to electrophilic xenobiotic compounds (Yu 1996; Kim and Yim 2013). The CYPs (EC 1.14.‐.‐) are a superfamily of hemoproteins that catalyze monooxygenase reactions, and which are involved in endogenous metabolism as well as in the metabolism of xenobiotics, including the metabolization of plant chemical defenses and insecticides (Scott 1999; Li et al. 2007).

How predictive is the expression of these genes in response to chemical stress? Do the different enzymatic systems respond similarly to immediate and delayed chemical stress? According to Dhar et al. (2013), AGR (anticipatory gene regulation) occurs when organisms use information about its present environment, to adaptively trigger gene expression in future environments (Dhar et al. 2013). In this study, we analyzed AGR in the context of the reproductive strategy in aphids (Hemiptera: Aphididae). Aphids typically reproduce by cyclic parthenogenesis, which include several asexual parthenogenetic generations followed by a single sexual one. During the asexual phase, the viviparous parthenogenesis in aphids is characterized by the telescoping of generations (i.e., females carry their daughters and their granddaughters embryos in the abdomen) (Simon et al. 2010). Hence, we evaluated whether gene expression of candidate genes related to detoxification could be regulated in mothers, but triggered in their daughters. To achieve this goal, we reared several generations of aphids (*Myzus persicae*) in bell peppers (*Capsicum annuum*), (the “suitable” host) which are known to have relatively low levels of defensive compounds, and in radish (*Raphanus sativus*), which is known to have several toxic compounds (Olivares‐Donoso et al. 2007). It is also established that susceptible genotypes perform significantly worst in radish, compared with peppers (Silva et al. 2012a,b). We then measured the relative abundance of transcripts encoding detoxifying enzymes in the daughters (see Methods). All experiments were performed only on insecticide susceptible genotypes (i.e., not carrying any insensitivity mutation; see (Silva et al. 2012a)), which show significant gene regulation in the four aforementioned enzymatic systems, either in response to insecticides or plant chemical defenses (David et al. 2010; Schuler 2012). We predicted that stressful environments experienced by mothers (i.e., growing in radish) induce a response (in terms of differential gene expression) in the daughters. More specific predictions are summarized in Table 1.

**Table 1:** The hypothetical levels of stress that aphids perceived according to our experimental design. Two generations of aphids were grown in a nonstressful (pepper) and a stressful host (radish), and gene expression was measured in the second generation (see Methods for details)

Levels of stress	Situation experienced		They can anticipate?
0 (no stress)	Mother raised in pepper	Offspring raised in pepper	Cannot anticipate
1 (mildly stressful)	Mother raised in pepper	Offspring raised in radish	Cannot anticipate
2 (stressful)	Mother raised in radish	Offspring raised in pepper	They can anticipate
3 (very stressful)	Mother raised in radish	Offspring raised in radish	They can anticipate

## Materials and Methods

### Collection sites, maintenance and microsatellite genotyping

Detailed information regarding sampling locations, maintenance and microsatellite genotyping can be found in Castaneda et al. (2011). In brief, 94 individual aphids were sampled next to roads and agricultural fields along an 1830 km latitudinal transect in Chile. Parthenogenetic lineages were separately established in Blackman box cages containing seedlings of *Capsicum annuum* var. Grossum from a single adult wingless female and maintained at 20 ± 1°C and LD 16:8, conditions that ensure the parthenogenetic reproduction of aphids. Every 10 days, five wingless adults were transferred to new seven‐day‐old pepper seedlings. Aphid lineages were reared on pepper seedlings for at least 20 generations before experiments.

Each aphid was genotyped at six previously described microsatellite loci (*Myz2, Myz3, Myz25, M35, M37, M40*) (references in Castaneda et al. 2011). In a total sample of 94 aphids, we could identify 44 different genotypes.

### Insecticide resistance assessment

In order to select only susceptible genotypes, the presence of IRM (insecticide resistance mutations) was screened among genotypes, using allelic discrimination based on quantitative‐PCR assays for *kdr* (L1014F) and super*‐kdr* (M918T) mutations (Anstead et al. 2004), and an analogous procedure for MACE mutation (Anstead et al. 2008) (see also Silva et al. 2012a,b for details). Thirty‐three of 44 genotypes were labeled as “sensitive” as they did not carry any resistance mutations. Six genotypes were heterozygote for *kdr,* and five were heterozygote for both *kdr* and MACE mutations. No genotype was found to carry either MACE or *kdr* mutations in homozygous state or carrying a super‐*kdr* mutation (Silva et al. 2012b). Seven sensitive genotypes were randomly selected and propagated for the experiments. No statistical differences between these seven genotypes were found in any of the traits measured in this study.

### Breeding design

In order to test the effects of the host plant on the reproductive fitness and gene expression of *M. persicae*, we carried out a design that comprised several generations on each host, using suitable hosts (bell pepper) and nonsuitable hosts (radish) (Olivares‐Donoso et al. 2007; Silva et al. 2012a).

One single adult wingless aphid (parental) from each selected genotype was transferred to a 3‐month‐old radish plant and left to reproduce during 24–48 h. Ten parthenogenetic nymphs were maintained in the same plant until adulthood, discarding the rest of the aphids, giving rise to ten clonal lines per genotype. Each of these aphids was then transferred to a new 3‐month‐old plant for erasing maternal and granmaternal host effects after three rounds of parthenogenetic reproduction on radish. F4 individuals from each clonal line per genotype were then transferred to a seven‐day‐old pepper plant, and maternal and granmaternal host effects of pepper were erased after three rounds of parthenogenetic reproduction in pepper. Five clonal lines per genotype of F8 individuals (i.e., the mothers) were maintained on pepper, while the other five were switched to radish. Five offspring (i.e., focal individuals, in which quantifications were performed) per mother were kept on the same host as their mothers, and five were switched to the alternative host.

With this design, the direct experience of the mothers could be tested in their daughters, in isolation to previous maternal and grand‐maternal experiences. Particularly, informative of AGR is the comparison between the “nonstressful” condition (mother and offspring raised in the nondefended host) and a “stressful” condition where the mother is raised in the defended host, but the offspring is transferred to the nondefended host (see our operational definitions in Table 1). Two kinds of variables were measured in these individuals, fitness and gene expression using RT‐qPCR. Given that aphids were utilized in the RT‐qPCR trials, the whole experiment was repeated for fitness assays. Fitness was measured for each focal individual whereas for RT‐qPCR, three biological replicates were taken from each pool of individuals per host (which came from the ten clonal replicates per each of the seven genotypes, see above). All these measurements were performed when aphids became adults, in whole bodies.

### Fitness assays

Fitness, measured as the intrinsic rate of natural increase (*r*_*m*_), was determined for all focal daughters accordingly to Wyatt and White (1977). In brief, for each individual, we determined the AFR (age at the first reproduction) and the ON (offspring number) generated in a given time lapse of parthenogenetic reproduction. For example, if an aphid had its first nymph 6 days after being born (i.e., AFR), we counted its progeny for 6 days (i.e., ON). Then, *r*_*m*_ was calculated as *r*_*m*_ = 0.74·(log_e_ ON)/AFR, where 0.74 is a correction factor (Wyatt and White 1977).

### Quantitative reverse‐transcription PCR (RT‐qPCR)

The transcriptional levels of four genes, cathepsin B‐N (Cath‐b), E4 esterase (Field and Devonshire 1998), GST (glutathione S‐transferase), and cytochrome p450 (CYP), were determined by RT‐qPCR in adult aphids (focal offsprings, according to Table 1) that passed 72 h in the host. These genes are known to be regulated after exposure to carbamate insecticides (especially in susceptible genotypes, see Silva et al. 2012a,b).

Aphids were collected from their plants and immediately frozen in liquid N_2_ until RNA extractions. Detailed methods can be found in Silva et al. (2012a,b).

In brief, total RNA was extracted using the RNeasy Plant Mini Kit (Qiagen, Cat no. 74904, Venlo, Nederlands) from three aphids per genotype and host. cDNA synthesis was prepared using AffinityScript QPCR cDNA Synthesis kit (Agilent, Santa Clara, California, USA). We included negative controls for detecting foreign contamination, being all PCRs performed in triplicate in an Mx3000P QPCR Systems (Stratagene, Agilent, Santa Clara, California, USA). Primers were designed from the sequences of *M. persicae* contigs for four target genes, Cath‐b (EC387286), esterase E4 (EE261252), GST (EC387215), CYP (cytochrome P450 4 g15‐like, EE263097), and also for endogenous control gene glyceraldehyde‐3‐phosphate dehydrogenase (DW011095). The primers were checked in NCBI/Primer‐BLAST (for details in primer sequences and PCR efficiencies, see Silva et al. 2012a,b).

The relative expression ratio of a target gene was computed by relative quantification using the comparative Ct method (Applied Biosystems User Bulletin No. 2 P/N 4303859, 1997) (Livak and Schmittgen 2001), with the GADPH (glyceraldehyde‐3‐phosphate dehydrogenase) and the actin mRNA genes as normalizing endogenous controls (see Silva et al. 2012b, for details) (Farcy et al. 2009).

### Statistical analysis

As analyses using both normalizing genes gave identical results, we only present the results using the GAPDH gene. We used a linear mixed modeling approach to evaluate the effect of maternal and offspring host on *r*_*m*_ and gene expression while taking into account the presence of random factors (genotype), the nested structure of our design (clonal lines were nested into genotypes), and some unbalance. Hypothesis testing for fixed effects was based on LRT (likelihood ratio tests) of nested models based on ML (maximum likelihood) estimation. We used a generalized mixed modeling approach with a logit link function to evaluate whether maternal and offspring host affected the survival of mothers, focal individual as nymphs, and focal individuals as adults. Statistical analyses were performed using the lme4 package implemented in R platform 3.0.2 (R Development Core Team, 2013).

## Results

**Figure 1:**
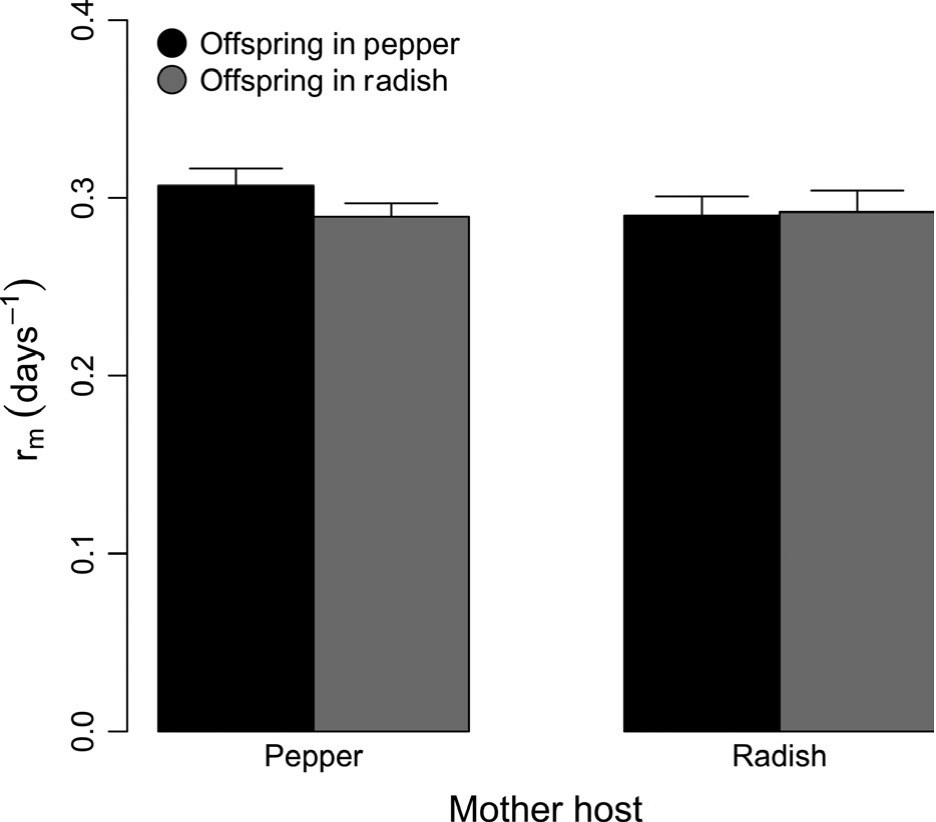
Intrinsic rate of natural increase (*r*_*m*_) for maternal and offspring hosts averaged across all seven susceptible genotypes (see Methods for details). Data are presented as mean ± 1SE.

In general, the fitness measured on offspring was not affected by the maternal host (*χ* ^*2*^_[1]_ = 0.962, *P* = 0.327), the offspring host (*χ* ^*2*^_[1]_ = 0.651, *P* = 0.420), or their interaction (*χ* ^*2*^_[1]_ = 0.745, *P* = 0.388) (Fig. 1). Maternal survival was not affected by the host in which they were reared (*χ* ^*2*^_[1]_ = 0.13, *P* = 0.717), and only 14 of 132 mothers died before producing any progeny (6 in pepper and 14 in radish). Furthermore, the number of focal individuals that died as nymph was also not affected by the host in which their mothers were reared (*χ* ^*2*^_[1]_ = 2.11, *P* = 0.146), the host where they were reared (*χ* ^*2*^_[1]_ = 2.02, *P* = 0.155) or their interaction (*χ* ^*2*^_[1]_ = 0.26, *P* = 0.608). In overall, 30 nymphs died in pepper and 42 in radish. On the other hand, the number of focal individuals that died as adults was affected by the host in which they were reared (*χ* ^*2*^_[1]_ = 23.06, *P* < 0.001), but not by their mother's host (*χ* ^*2*^_[1]_ = 0.96, *P* = 0.327) or interaction (*χ* ^*2*^_[1]_ = 0.01, *P* = 0.795). In particular, 5 individuals died in pepper while 30 died in radish. In summary, daughter survival appears not to be affected by the host were mothers grew.

**Figure 2:**
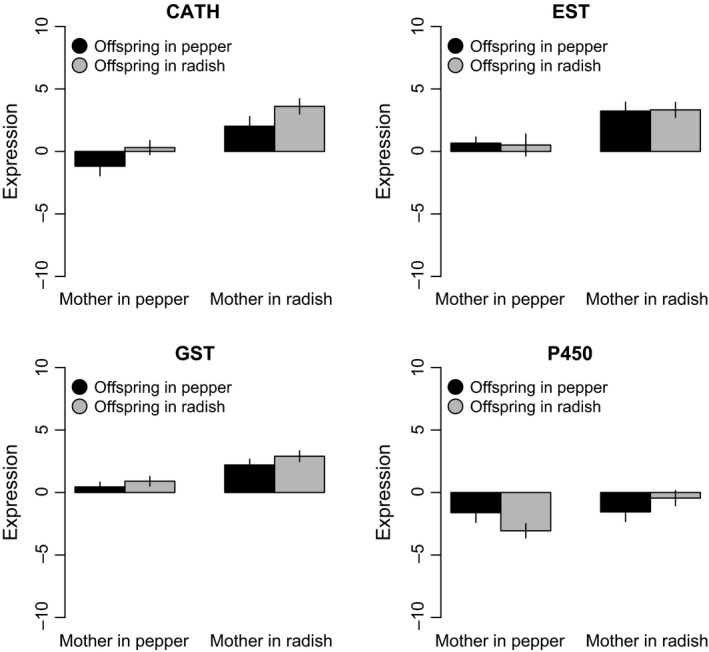
Expression levels of four genes that participate in xenobiotic detoxification in aphids reared in either a beneficial (i.e., pepper) or stressful (i.e., radish) host, and that come from mothers that were also raised in either host (i.e., two generations in the same host, or suffered a host shift in the second generation). Data are presented as mean ± 1SE of seven genotypes that are known to be susceptible for allelochemicals (see Methods for details). CATH refers to cathepsin B; GST refers to glutathione S‐transferase; esterases refers to esterase E4/FE4; and CYP refers to cytochrome P‐450 families (see Methods for details). Values correspond to levels of mRNA of each studied gene standardized to GADPH, as normalizing gene (see Farcy et al. 2009, and Methods, for details).

We found variable effects of maternal and offspring hosts on the gene expression of detoxifying genes. Both the offspring (*χ* ^*2*^_[1]_ = 20.11, *P *<* *0.001) and maternal (*χ* ^*2*^_[1]_ = 4.87, *P *=* *0.027) hosts affected the expression of Cath‐b: in both cases, the gene expression was higher in radish (Fig. 2). In this case, the data support a “completely additive” prediction: (3) > (2) > (1) > (0) (see Table 1). Nevertheless, the interaction between both factors was not significant (*χ* ^*2*^_[1]_ = 0.0004, *P* = 0.985). The expression of EST and GST was higher when offspring were raised on radish (EST: *χ* ^*2*^_[1]_ = 9.95, *P *<* *0.01; GST: *χ* ^*2*^_[1]_ = 23.34, *P *<* *0.01) (Fig. 2). However, we detected nonsignificant effects of the maternal host (EST: *χ* ^*2*^_[1]_ = 0.03, *P *=* *0.854; GST: *χ* ^*2*^_[1]_ = 2.36, *P *=* *0.124) or the interaction among them on the expression of EST (MH x OH: *χ* ^*2*^_[1]_ = 0.07, *P* = 0.795) or GST (MH x OH: *χ* ^*2*^_[1]_ = 0.21, *P* = 0.651) (Fig. 2). Thus, in terms of our predictions (Table 1), for EST and GST responses were (3) = (2) > (1) = (0) (see Table 1). Finally, the expression of the CYPs was not significantly affected by the maternal host (*χ* ^*2*^_[1]_ = 0.12, *P* = 0.727), offspring host (*χ* ^*2*^_[1]_ = 2.62, *P* = 0.106), or their interaction (*χ* ^*2*^_[1]_ = 3.28, *P* = 0.070) (Fig. 2), suggesting no anticipatory response for these detoxifying genes.

## Discussion

One of the most important challenges of evolutionary theory is to understand the mechanisms linking population dynamics with adaptive evolution, which has proven to be extremely difficult (Nespolo 2007; Zehnder and Hunter 2007; Coulson et al. 2011). A key element in this task is to characterize the rules by which environmental cues determine differential gene expression, and how this in turn impact fitness. In this study, we found that environmental heterogeneity (in terms of the quality of the host) could trigger anticipated gene expression (in terms of the abundance of generalized transcripts for a given enzyme system) for attributes that impact performance (in terms of survival under stressful conditions). In particular, we found that the time‐course of gene regulation varied in different detoxifying systems. Cathepsins, which are dietary proteases involved in xenobiotic metabolizing (but also in nutrient digestion), seem to react more gradually across generations, whereas esterases and GSTs (enzymes hydrolyzing inorganic compounds) seem to show the maximum expression, which is maintained even when the stimulus is mitigated, in a second generation. However, in the case of CYPs (monooxygenases involved in a variety of reactions, associated with electron transfer chains), no response was detected. Interestingly, these large and varied regulatory responses did not translate into fitness differences. In fact, differences in fitness between hosts were not statistically detectable at any level (i.e., measured in focal individuals or their mothers). With some caveats, this could be interpreted that the adaptation is being maintained across hosts.

There are many examples of compensatory changes at the biochemical or physiological level that maintains fitness under temporally and spatially variable environments (Castaneda et al. 2010; Bell and Gonzalez 2011; Dhar et al. 2011). For instance, plants can maintain fitness through compensatory biomass reallocation (Puijalon et al. 2005), vertebrates can maintain fitness by physiological flexibility in gut‐processing enzymes (Cortes et al. 2011), and yeasts can maintain fitness under salt stress by differential gene expression (Dhar et al. 2011). In fact, aphids can use the same set of genes to exhibit several environmentally cued polyphenisms (i.e., discrete alternative phenotypes) such as wing and reproductive polyphenisms due to population density and seasonal photoperiodism, respectively (Le Trionnaire et al. 2008). In this sense, our results are partially in agreement with what Loayza‐Muro et al. (2000) reported in *Sitobion avenae* exposed during ten generations to defended and nondefended plants. These authors found an intergeneration increase in four of five studied detoxifying enzymes, which was interpreted as adaptive plasticity that permit aphids to be prepared to face more toxins (Loayza‐Muro et al. 2000).

It is known that Solanaceae and Brassicaceae plants contain protease inhibitors that can be neutralized by insect herbivores through the action of cathepsins (Jamal et al. 2013). Hence, Cath‐b was up‐regulated between generations to compensate those that are being inhibited, which would explain the gradual up‐regulation for this gene (particularly observed when aphids are reared on a nonsuitable host). CYP, on the contrary, showed a more rigid pattern. The CYP gene tested was *CYP6CY3*, a gene that is found in multiple copies and constitutively highly overexpressed (up to 22‐fold) in *M. persicae* (Puinean et al. 2010). Hence, it is possible that the rigidity in the expression of this gene (under the context of susceptible genotypes) can be explained by gene amplification rather than gene regulation, which is expected to be independent of the treatment (e.g., different host plants, insecticides).

In general, our results suggest that previous experience of the mother seems to have a key role on the regulation of ESTs and GSTs in its progeny. Indeed, when aphids are reared on the nonsuitable host, the progeny is able to up‐regulate these detoxifying genes independently of its rearing host (suitable or nonsuitable), while no regulation is evidenced in the progeny when their mothers are reared on a suitable host. It is possible that trans‐generational developmental regulation through viviparity would explain the observed differences for these two detoxifying genes. Several authors have documented trans‐generational environmental effects in insects. For instance, Cahenzli and Erhardt (2013) showed, by a similar experiment as in this study (i.e., raising parents and offspring in contrasting hosts), that butterflies could adjust progeny's phenotype in function of the type of host they experience (Zehnder and Hunter 2007; Vorburger et al. 2008). Although our results show the existence of this capacity, and its timing and specificity, further research is certainly needed to determine the generality of such response and its molecular mechanism, not only in susceptible genotypes. It is known, for instance, that microRNAs are involved in the environmental induction of gene‐regulatory changes of generalist insects (Freitak et al. 2012). Whether other epigenetic marks, such as histone modifications and DNA methylations (see Collotta et al. 2013), are also important is unknown yet.

In summary, we think our results are interesting because they show how dynamic adaptive evolution is, even when fitness show no changes. Animals seem to be constantly on the move to track their environment, experiencing subtle adjustments in gene expression that maintain the adaptation. We believe these are exciting avenues for research that will improve our understanding how genes, transcripts, and fitness interact in populations when facing highly dynamic evolutionary landscapes.

## Conflict of Interest

None declared.
